# The gene expression of *Leishmania infantum chagasi* inside *Lutzomyia longipalpis*, the main vector of visceral leishmaniasis in Brazil

**DOI:** 10.1590/0074-02760200571

**Published:** 2021-03-08

**Authors:** Thais Lemos-Silva, Erich Loza Telleria, Yara Maria Traub-Csekö

**Affiliations:** 1 Fundação Oswaldo Cruz-Fiocruz, Instituto Oswaldo Cruz, Laboratório de Biologia Molecular de Parasitas e Vetores, Rio de Janeiro, RJ, Brasil; 2 Charles University, Faculty of Science, Department of Parasitology, Prague, Czech Republic

**Keywords:** *Leishmania*-sand fly interaction, parasite adaptation to vector, sand fly gut environment

## Abstract

*Leishmania infantum chagasi* is the causative agent and *Lutzomyia longipalpis* is the main vector of visceral leishmaniasis in the Americas. We investigated the expression of *Leishmania* genes within *L. longipalpis* after artificial infection. mRNAs from genes involved in sugar and amino acid metabolism were upregulated at times of high parasite proliferation inside the insect. mRNAs from genes involved in metacyclogenesis had higher expression in late stages of infection. Other modulated genes of interest were involved in immunomodulation, purine salvage pathway and protein recycling. These data reveal aspects of the adaptation of the parasite to the microenvironment of the vector gut and reflect the preparation for infection in the vertebrate.

The journey of *Leishmania* within the phlebotomine sand fly vector involves many adaptive processes for ensuring parasite survival. In the early phase of infection in the sand fly, the ingested blood goes through digestive processes with increased enzymatic activity and oxidative stress, creating a hostile environment for the parasite in the insect gut. Another crucial event occurs after blood digestion when parasites must migrate to the anterior midgut and adhere to the intestinal epithelium, therefore avoiding being excreted together with the digested blood. The insect immune system is active during the entire infection cycle and becomes one additional barrier faced by *Leishmania* inside the sand fly.^1^ To overcome all these challenges the parasite goes through fundamental steps of morphological differentiation requiring the expression of specific molecules.

Studies of *Leishmania* gene expression on parasites obtained from culture, isolated from host cells or from sand fly vectors were performed to understand its survival mechanisms,^2^ but few of them investigated natural parasite-vector pairs.^3^ ^,^ ^4^ In the case of the *Leishmania infantum* and *Lutzomyia longipalpis* natural pair, Coutinho-Abreu et al.^4^ performed a transcriptomic analysis of the parasite genes expressed within *L. longipalpis* at different times post infection (PI), and highlighted genes that could be used as putative stage-specific molecular markers for *L. infantum*. In the present work we pinpointed *Leishmania* genes potentially involved in interaction with the insect gut, parasite differentiation and proliferation, and modulation of host immune response. Under this context, we investigated the expression of these genes along the development of infection in the insect vector. We emphasise that the current studies only monitor changes in levels of mRNAs during development of the parasite within the insect. Regulation at post-mRNA levels, such as translation into protein or post-translational modifications, could either amplify or negate the effects of changes in mRNA levels observed here.

We performed quantitative polymerase chain reaction (qPCR) to study the expression of *L. infantum* selected genes using specific primers and cDNA samples obtained from pools of whole *L. longipalpis* females (colony originally from Jacobina, Bahia, Brazil) fed on defibrinated rabbit blood containing 1 x 10^7^ *L. infantum chagasi* (syn. *L. infantum*) promastigotes/mL (MHOM/BR/1974/PP75) as described by Di-Blasi et al.^5^ All experiments were performed using at least three biological replicates. Relative gene expression was calculated using the ΔΔCT method. The expression was normalised using *Leishmania* actin as reference gene, and infected sand flies collected at 1 h PI were used as the control sample. Reference and target gene primers are described in the Table. qPCR was performed under the same cycling conditions described by Di-Blasi et al.^5^ The statistical analyses were performed using the GraphPad Prism 6 software version 6.01 (GraphPad Software, Inc.).

**TABLE:** Reference and target genes primers used in this work

Gene	Foward primer sequence	Reverse primer sequence	Gene ID or Reference
Actin	GTCGTCGATAAAGCCGAAGGTGGTT	TTGGGCCAGACTCGTCGTACTCGCT	Di-Blasi et al.^9^
Amino acid permease 3 (AAP3)	GCCTACCACTGCCTGAACT	GATGCTCGGGATGAACAGA	LINF_310014600; LINF_310014500
Chitinase (CHIT1)	ACAAGCGTTCATAGAGGAGGT	CAGCCACTCCGTCATTGTTT	LINF_160013400
Elongation factor 1 alpha (EF1-alpha)	CCCTCCCTCCACCCTTT	CGCGCACACGCATATATAGAA	LINF_170006100
Flagellar protein FLAG1/SMP1	AGTGGGTAGCCTCCGTGGTGGTGTA	CTCCGACAGCGGCAAGGCGTCCATC	Di-Blasi et al.^9^
Glucose transporter 1 (GT1)	AGGCTCCCTCATAATGTGCT	AATCTGGTGCGGTGTCTTC	LINF_360073600
Glucose transporter 2 (GT2)	ACGCCAGGATGCAAAGAA	ATGGCCTGCACCAACATAA	LINF_360073200
Glucose transporters 1, 2 and 3 (GTs)	CAAGAAGACGGAGGTGAAGAA	CCTGCGTCAGAACGTAGTAG	LINF_360072800; LINF_360073200; LINF_360073600
Kinetoplastid membrane protein-11 (KMP-11)	AAGCTGGACCGCCTGGAT	CGTAGTGCTCCTTCATCTCG	LINF_350027400
Leishmanolysin (GP63)	TCCTGGTCAAGCACCTCATC	TGCCCGTCACCTTCCACT	LINF_100010100; LINF_100011300; LINF_100010200; LINF_100011200
Peroxidoxin	ATCCGATGGACTTCACCTT	ACGACACCGCAACAACC	LINF_230005400
Polyubiquitin / Ubiquitin-fusion protein	AACTGGGAGAAGAAGGTGTG	GCAGGTTGGAGCAGTGA	LINF_090015200; LINF_310026800; LINF_310028300
Small hydrophilic endoplasmic reticulum-associated protein (SHERP)	CCAGGAGACAAAGGACCAGA	AGCCTTATTGCTCACACCGTCCTTC	LINF_230018650; LINF_230018800
Xanthine phosphoribosyltransferase (XPRT)	ATCTTCACATCCGACTTCATCC	GCTCTTTGTACCCGAGTTGT	LINF_210015000

*Interaction with the insect gut* - *Leishmania* expresses chitinase, a glycoside hydrolase, in the insect gut, causing the degradation of chitin-rich surface of the sand fly stomodeal valve. This degradation reduces the efficiency of the valve in retaining the gut content and facilitates parasite reflux at the bite site.^6^ In *Leishmania mexicana*, the chitinase gene is constitutively expressed in the procyclic and metacyclic-like promastigotes from culture and amastigote forms of the parasite.^7^ In *L. i. chagasi* we observed that the chitinase gene had no significant differential expression during infection in *L. longipalpis*, although there was a tendency for increase in late times PI (Fig. 1A). It is possible that this slight increase may be correlated with the initial parasite colonisation and consequent degradation of the sand fly stomodeal valve.

**Fig. 1:**
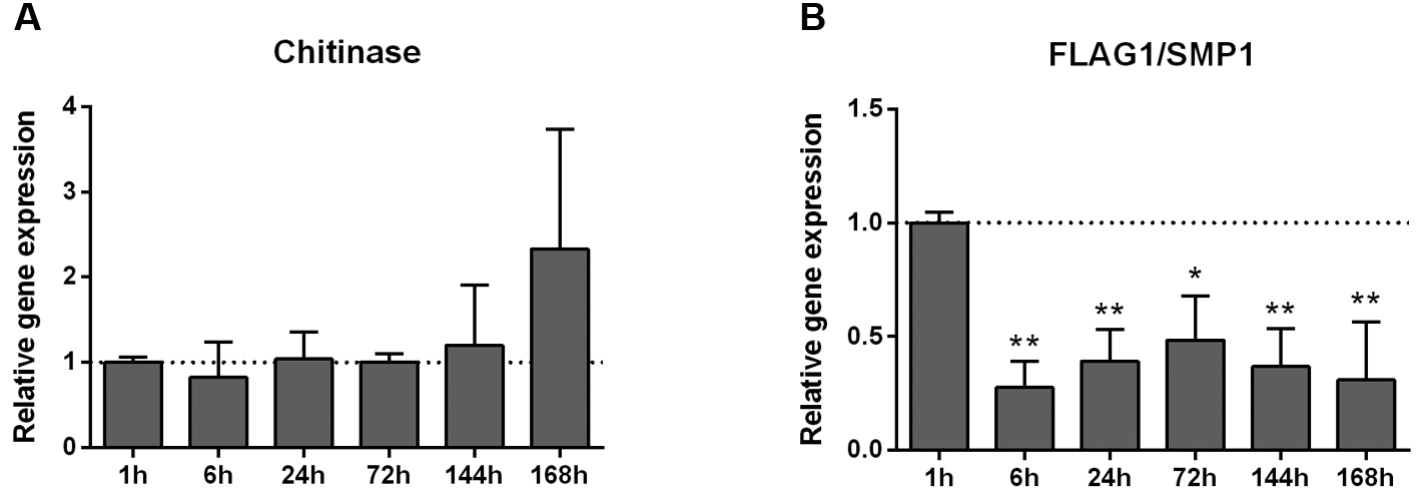
expression of genes involved in the interaction with the gut of the insect. Analysis of variance (ANOVA) tests with Tukey multiple comparisons post-test (*p < 0.05; **p < 0.01).

The parasite flagellum participates in the adhesion to the sand fly gut through the formation of hemidesmosomes with the cuticular lining of the stomodeal valve, and insertion of the flagellum between microvilli of midgut cells.^8^ Di-Blasi et al.^9^ showed that the flagellar protein FLAG1/SMP1, from the family of Small Myristoylated Proteins (SMPs), has a role in infection depending on the parasite and vector pair. In the *Leishmania major* and *Phlebotomus papatasi* pair, the FLAG1/SMP1 protein of the parasite was important for interaction with the midgut and for a successful infection. Recent studies described SMP-3 as a virulence factor, stimulating the vertebrate Th1 immune response in *Leishmania amazonensis*, *L. infantum* and *Leishmania braziliensis* infections.^10^ Here we investigated the gene expression of FLAG1/SMP1 during infection in the vector and the gene expression was reduced after 1h PI (Fig. 1B). The downregulation of FLAG/SMP1 after the parasite development in the sand fly gut adds to Di-Blasi et al.^9^ results showing that this molecule is not crucial for the interaction between *L. longipalpis* and *L. i. chagasi* and does not seem to work as a virulence factor during vector infection.

*Parasite nutrient acquisition, differentiation and proliferation* - The *Leishmania* life cycle in the insect gut includes various morphological changes. Some forms have intense proliferation (procyclic and leptomonads) while others do not divide (elongated nectomonads and metacyclics).^11^ Throughout their heteroxenic life cycle *Leishmania* parasites adapted to different energetic sources. Amastigotes are exposed to low concentrations of carbohydrates within the macrophage phagolysosome and their main nutrient sources are fatty acids and amino acids. Promastigotes, however, are exposed to high concentrations of carbohydrates acquired by the sand fly from blood and plants sap, from which the insect obtains glucose and fructose.^12^ We selected several genes involved in parasite nutrient acquisition, cell differentiation and proliferation to explore their expression.

There are three *Leishmania* glucose transporters (GTs), named GT1, GT2 and GT3. GT1 is expressed only on the parasite flagellum, whereas GT2 and GT3 are expressed only on the parasite’s cell body membrane. In addition to glucose, GTs also have a role in the uptake of fructose, mannose and galactose, and GT1 participates as a sensor responding to glucose levels, acting on the adaptation of the parasite to environmental changes.^13^

We analysed GT1 and GT2 expression, and since the GT3 gene is very conserved in comparison to the other GTs it was impossible to design qPCR specific primers for this gene, therefore conserved primers were designed that cover the three GTs (herein mentioned as overall GTs expression) (Fig. 2A-C). Our results showed a similar pattern of expression among *L. i. chagasi* GTs with a progressive decline in expression after 6 h and 144 h (Fig. 2A-C). This downregulation matches the surge of non-replicating forms of the parasite and may be related to a switch on their nutritional uptake.^14^ Coutinho-Abreu et al.^4^ in their supplementary data also showed a downregulation of *L. infantum* GT2 and GT3 in early infection stages. However, this profile is different from what was observed in *L. major* GTs within *Phlebotomus duboscqi* where the expression is higher in late times PI.^3^ Such distinction may occur in response to how these two parasite species adapt to the sand fly vectors, or differences in sugar metabolism.

**Fig. 2:**
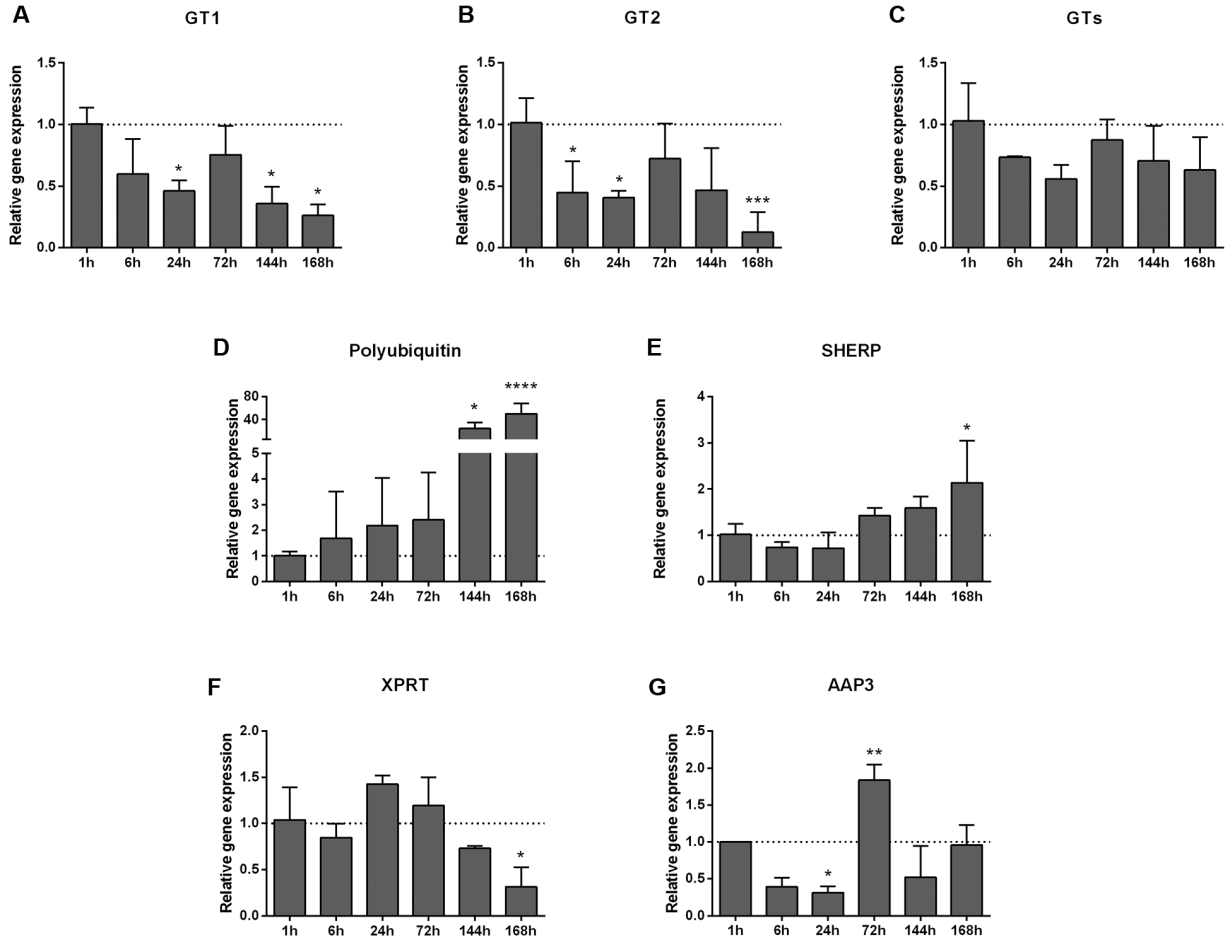
expression of genes involved in parasite differentiation and proliferation. Analysis of variance (ANOVA) tests with Tukey multiple comparisons post-test (*p < 0.05; **p < 0.01; ***p < 0.001; ****p < 0.0001).

Besides carbohydrates, proteins are indispensable sources of amino acids for building cellular structure and for metabolism, and *Leishmania* parasites developed several ways to acquire and recycle these valuable nutrients. For instance, the classic ubiquitin-proteasome system is responsible for protein recycling and is present universally in eukaryotes. Its action consists of tagging target proteins with ubiquitin molecules forming polyubiquitin chains, which are recognised and degraded by the proteasome. In protozoan parasites, this system is crucial in cell differentiation and proliferation.^15^ The importance of the ubiquitin-proteasome system in *Leishmania* was demonstrated through the proteasome inhibition in promastigote and amastigote forms of the parasite, leading to loss of growth and replication capacity.^16^ In the present study, we observed a peak of *L. i. chagasi* polyubiquitin gene expression at 144 h and 168 h PI (Fig. 2D). It is important to highlight that inside the insect the metacyclic forms have the most remarkable morphological change resulting in longer flagellum and reduced cellular body when compared to procyclic forms. Therefore, it is possible that the increase in polyubiquitin expression is related to the parasite morphological remodeling inside the vector. Interestingly, a polyubiquitin mRNA was downregulated and a ubiquitin fusion degradation protein was upregulated in *L. infantum* procyclic promastigotes in comparison to nectomonads in the work done by Coutinho-Abreu et al.,^4^ thus reflecting a complex balance of molecules involved in protein recycling.

The small hydrophilic endoplasmic reticulum-associated protein (SHERP) is another molecule involved in parasite metacyclogenesis and important for parasite transmission to the vertebrate host.^17^ ^,^ ^18^ SHERP is present in the endoplasmic reticulum and the outer mitochondrial membrane and is possibly involved in parasite membrane organisation and acidification.^19^ Doehl et al.^18^ saw that *L. major* with deleted SHERP had stalled metacyclogenesis, failed to secrete promastigote secretory gel (PSG) and to colonise the anterior midgut in sand flies. In the present work, the expression of SHERP was increased at 168 h PI (Fig. 2E), consistent with its importance in late parasite development. Our results are in agreement with the expression of SHERP in two transcriptome studies that show *L. major* and *L. infantum* increased expression of SHERP inside their respective natural vectors *P. duboscqi* and *L. longipalpis* in late infection phase.^3^ ^,^ ^4^

Parasitic protozoa are auxotrophic for purines and must rescue purines from the host for survival. *Leishmania* xanthine phosphoribosyltransferase (XPRT) in combination with hypoxanthine-guanine phosphoribosyltransferase are essential in the purine acquisition pathway.^20^ The deletion of both genes was lethal for *Leishmania donovani*.^20^ In addition, Carter et al.^21^ showed that *L. donovani* XPRT gene expression was increased in an *in vitro* purine-deficient environment. We observed that the *L. i. chagasi* XPRT gene expression was reduced only at 168 h PI in *L. longipalpis* (Fig. 2F). In Coutinho-Abreu et al.,^4^ a XPRT mRNA was also downregulated in metacyclics when compared to leptomonads. It is possible that at late times post blood meal the insect gut environment might contain lower concentration of purines. Nevertheless, non-replicating haptomonad and metacyclic *Leishmania* forms, which most probably have a reduced demand for purines, become abundant at this point^11^ in comparison^22^ to the replicating forms used in *in vitro* assays.

The amino acid permease 3 (AAP3) is an amino acid transporter that has a high affinity for arginine. In the vertebrate host this transporter also acts in the homeostasis of arginine intracellular levels avoiding host immune pathways activation.^23^ AAP3 is involved in the polyamine pathway, which is crucial for parasite replication.^24^ During insect infection, *Leishmania* AAP3 presented a reduced expression at 24 h (Fig. 2G) a time point when *L. longipalpis* digestive trypsin reaches the highest activity^25^ and by extension digested nutrients are most available. Conversely, a greater AAP3 expression was observed at 72 h PI (Fig. 2G) when the sand fly trypsin activity is reduced, and blood digestion process is ending. This is similar to what was identified by Inbar et al.^3^ where *L. major* expressed more the AAP3 gene in nectomonad and metacyclic forms in *P. duboscqi*, probably associated with stress conditions and amino acid starvation. In the work done by Coutinho-Abreu et al.,^4^ two other amino acid permease were up-regulated in metacyclic stages. This corroborates the idea that in these moments the parasite increases its nutritional uptake and indicates conserved function across different vector-parasite pairs.

*Virulence and host immune response modulation* - *Leishmania* parasites thrive inside the macrophage, one of the most active defense cells of the vertebrate host. At the same time, these parasites establish their cycle in the sand fly vector resisting to several insect immune mechanisms. Interestingly, insect immunity shares some similarities with the vertebrate immune response.^1^

The parasite is susceptible to oxidative stress and peroxidoxin plays an important role against it by acting in the detoxification of peroxides. Although *Leishmania* infection does not increase reactive oxygen species (ROS) levels in the sand fly gut, promastigotes come in contact with the already existing reactive oxygen species generated during blood digestion.^26^ We evaluated the gene expression of *L. i. chagasi* peroxidoxin during the parasite establishment in *L. longipalpis* and observed that the expression of this gene is increased at 168 h (Fig. 3A). This result is in agreement with the up regulation of proteins involved in resistance to oxidative stress seen in metacyclic-like promastigotes of *L. amazonensis*,^27^ indicating a possible role for these genes in the parasite adaptation to the insect gut.

**Fig. 3:**
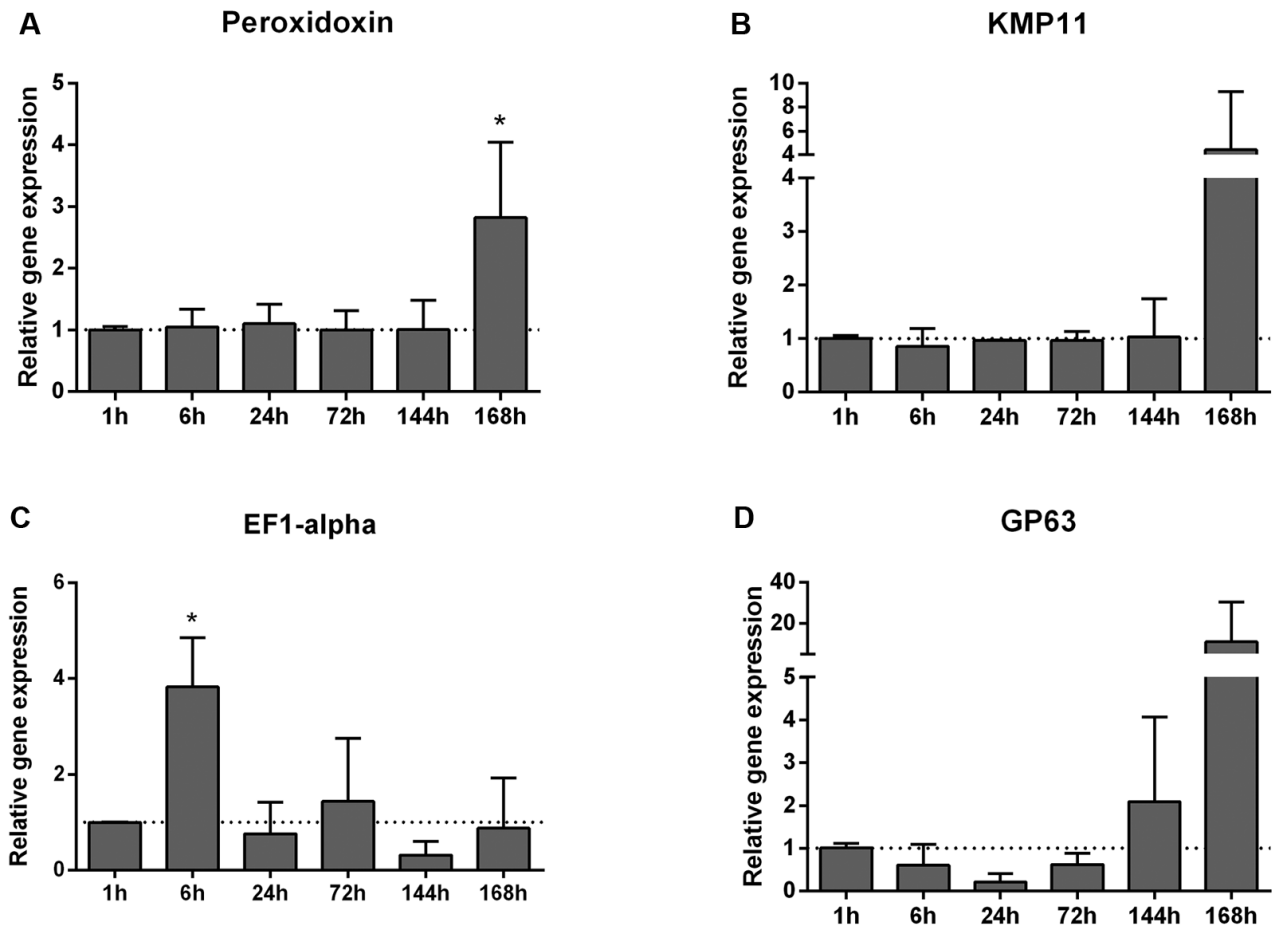
expression of genes involved in virulence, resistance, and host immune response modulation. Analysis of variance (ANOVA) tests with Tukey multiple comparisons post-test (*p < 0.05).

We also investigated the gene expression of molecules involved in modulating the host immune response. The *Leishmania* kinetoplastid membrane protein-11 (KMP-11) is found in association with membrane structures, such as the cell surface, flagellar pocket, and intracellular vesicles. This protein was more expressed in infective forms such as late culture metacyclic-like promastigotes and amastigotes.^28^ In addition, this protein has the ability to stimulate the production of interleukin 10 that acts on the macrophage immunosuppression in the vertebrate host.^29^ We did not see a significant modulation of KMP-11 at the transcriptional level by *L. i. chagasi* infecting *L. longipalpis* (Fig. 3B). Although KMP-11 has an important role for infection in the vertebrate host, additional studies would be required to draw any hypothesis on its role in the vector.

The elongation factor 1-alpha (EF1-alpha) is a GTPase involved in the polypeptide regulation of translation. In *Leishmania* parasite, EF1-alpha also is related to the binding and activation of the macrophage tyrosine phosphatase 1 (SHP-1) that acts as a repressor of the Toll and Jak/STAT pathways, immunosuppressing the host.^30^ Interestingly, in the present work EF1-alfa was more expressed 6 h PI (Fig. 3C), and this increase in expression may be directly associated to the virulence factors abundance seen in *L. i. chagasi* promastigotes exosomes.^31^ This indicates that this molecule may be involved in the initial process of establishing infection in the invertebrate host, drawing our attention to the similarities between the mechanisms used by the parasite to highjack both macrophage and sand fly immune responses.

GP63 is a widely studied *Leishmania* molecule and much is known about its importance during infection of the vertebrate host. In a study of infection of *L. major* knockout for GP63 in *P. duboscqi*, no difference was detected in the survival of *Leishmania* when compared to the control wildtype.^32^ However, *L. amazonensis* underexpressing GP63 in *L. longipalpis* had reduced parasite survival at initial times of infection.^33^ In addition, GP63 is also important for the *L. infantum* and *L. braziliensis* attachment to the gut of their respective vectors *L. longipalpis* and *L. intermedia*.^34^ Furthermore, Inbar et al.^3^ and Coutinho-Abreu et al.^4^ observed that *L. major* and *L. i. chagasi* expressed more GP63 in the late forms within the invertebrate host. Similarly, in the present work we show that the GP63 gene expression is increased in the late hours PI (Fig. 3D). These findings suggest that, in addition to a possible function in the modulation of the insect immunity, parasites may be preparing for contact with the vertebrate host while they are still inside the insect vector.

In conclusion, we have pinpointed *Leishmania* genes with a potential role in parasite-phlebotomine interaction and raised possible explanations on how these genes could play a role in the complex sand fly gut environment, although we cannot rule out other biological events involved. We observed that GT1 and GT2 which are involved in sugar metabolism were downregulated at times when non-replicative forms of the parasite start to appear. Conversely, AAP3 which is involved in amino acids metabolism is upregulated when there is high parasite proliferation inside the insect. SHERP and GP63 that are important in the metacyclogenesis in the invertebrate host and invasion of the macrophages in the vertebrate host, have higher expression in late stages of infection in the insect. EF1-alpha is upregulated in the early times of infection in the insect and could be important also in invertebrate immunomodulation. XPRT that is important in the purine salvage pathway has reduced expression at late times of infection. Polyubiquitin, which participates in ubiquitin-proteasome system responsible to recycle proteins, was upregulated at late times of infection and could be related with morphological parasite change to metacyclic form. Altogether, these data showed aspects of the adaptation of the parasite to the microenvironment of the vector gut and preparation for infection in the vertebrate.
